# Integrated Knowledge Translation for Non-Communicable Diseases: Stories from Sub-Saharan Africa

**DOI:** 10.5334/aogh.4228

**Published:** 2023-12-08

**Authors:** Nasreen S. Jessani, Peter Delobelle, Bonny Enock Balugaba, Talitha Louisa Mpando, Firaol Mesfin Ayele, Seleman Ntawuyirushintege, Anke Rohwer

**Affiliations:** 1 Stellenbosch University and the Institute of Development Studies, UK; 2 Chronic Disease Initiative for Africa, University of Cape Town, South Africa; 3 Department of Public Health, Vrije Universiteit Brussel, Belgium; 4 Department of Disease control and Environmental Health, Makerere University School of Public Health, Uganda; 5 School of Global and Public Health. Kamuzu University of Health Sciences, Malawi; 6 Non-communicable Disease Research Directorate, Armauer Hansen Research Institute, Addis Ababa, Ethiopia; 7 School of Public Health of the University of Rwanda; 8 Centre for Evidence-Based Health Care, Stellenbosch University, South Africa

**Keywords:** evidence-informed decision-making, integrated knowledge translation, citizen engagement, non-communicable disease, South Africa, Malawi, Ethiopia, Rwanda, Uganda, evidence use, network, stakeholder engagement

## Abstract

Integrated Knowledge Translation (IKT) is a key strategy for contextualising, tailoring, and communicating research for policy and practice. In this viewpoint, we provide examples of how partners from five countries in sub-Saharan Africa used IKT to advance interventions for curbing non-communicable diseases in their contexts and how these strategies were magnified during the COVID-19 pandemic in some cases. The stories highlight the importance of deliberate and reinforced capacity building, authentic relationship enhancement, adaptable and user-informed stakeholder engagement, and agile multi-sectoral involvement.

## Background

Bringing evidence into policy and practice is complex. Structural, cultural, and political factors play a major role in the actual decision-making process. Furthermore, there is often a disconnect between the evidence and decision-maker needs. Integrated knowledge translation (IKT) is an integral cog in the wheel of evidence-informed decision-making (EIDM). It focuses on continuous engagement between researchers and decision-makers and aims to increase the uptake of evidence into policy and practice. Embedding an IKT approach was critical to the Collaboration for Evidence-Based Healthcare and Public Health in Africa (CEBHA+), which conducted primary research and evidence syntheses on preventing and treating non-communicable diseases (NCDs), particularly diabetes, hypertension, and road traffic injuries (RTIs).

The CEBHA+ IKT approach included strengthening capacity in IKT through training for all CEBHA+ partners in two short courses offered through Stellenbosch University’s Centre for Evidence-Based Health Care. These courses resulted in CEBHA+ teams from Malawi, Ethiopia, South Africa, Rwanda, and Uganda identifying key stakeholders and creating, revisiting and revising country-specific stakeholder engagement strategies. Using issue briefs to communicate with decision-makers was a key output from the training, which was complemented with support and mentorship throughout the life of the collaboration.

**Figure 1:**
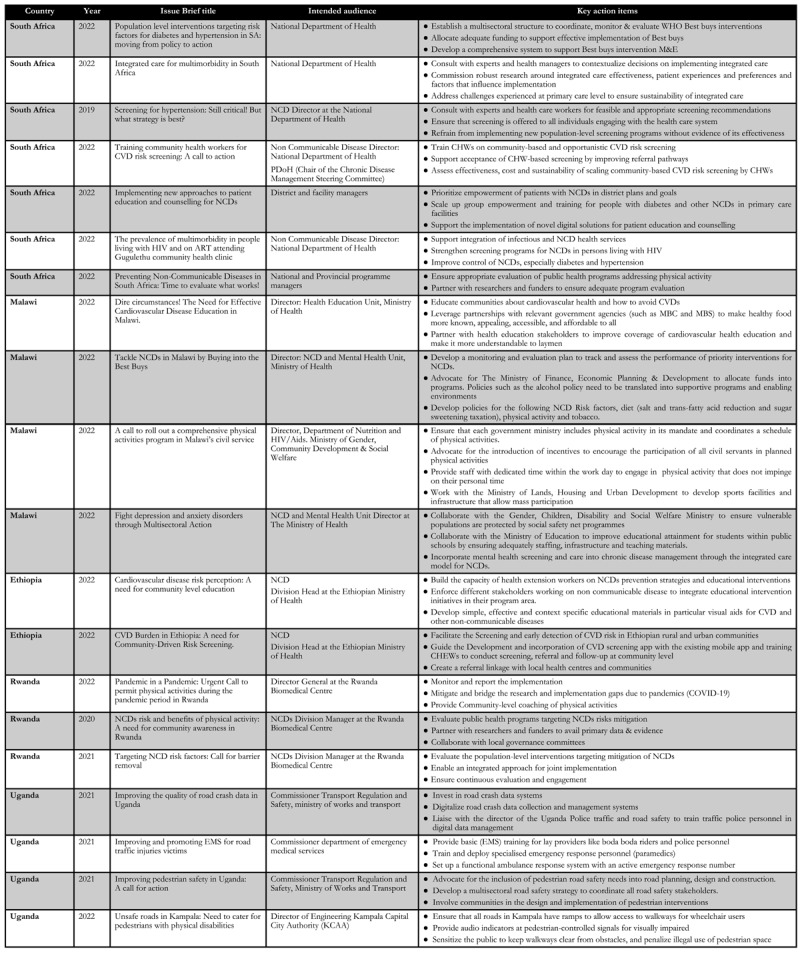
Summary of Issue briefs across the five African partners.

In this viewpoint, we aim to contribute to the discussions on stakeholder relationships and engagement as the cornerstone for advancing EIDM through illustrative stories. We draw on CEBHA+ IKT journeys from a spirited virtual storytelling panel discussion at the Consortium of Universities in Global Health conference held at Stellenbosch University in 2022. We report a short illustrative story from each country and present a summary of issue briefs in Figure 1.

In South Africa, Stellenbosch University, Cochrane South Africa, and the Chronic Disease Initiative for Africa coordinated primary research around cardiovascular disease (CVD) risk perception in CEBHA+ partner countries [1,2]; population-level interventions targeting risk factors of hypertension and diabetes; prevalence of diabetes among HIV-positive people; and comprehensive patient education and counselling in South Africa [3,4,5,6]; and evidence syntheses on screening [7,8], integrated models of care [9], and policy and regulatory interventions to prevent CVD and diabetes [10]. Evidence was shared with policy-makers using structured and ad hoc stakeholder engagement, capitalising on opportunities for personal feedback and discussion. Results were disseminated using different media and message formats and an NCD symposium convened in collaboration with the National Department of Health, where the Minister of Health stressed the importance of the role of EIDM. Seven issue briefs were produced. and discussed with national and provincial NCD representatives at a policy dialogue in November 2022.

In Uganda, where RTIs exert a heavy toll on pedestrians and people living with disabilities (PLWD) in particular, CEBHA+ researchers at the Makerere University Trauma, Injuries and Disabilities Unit undertook a desk review [11] and engaged road safety auditors to conduct a pedestrian road safety review and disseminated the findings among stakeholders. Using data on road crashes and pedestrian accidents from police and hospital records [12], stakeholder meetings were held with the Ministry of Works and Transport, Kampala Capital City Authority, members of Parliament, civil society, and the Uganda Ministry of Gender, Labour and Social Development. Issue briefs were developed and shared, and a symposium was hosted to address the daily mobility challenges faced by PWLDs. One of the panellists was a visually impaired person, which humanised the statistics and created momentum amongst decision-makers. Recognition of the challenges faced by vulnerable citizens resulted in media interest, thereby accelerating discussions with authorities on the budget allocation for interventions designed for PLWDs.

The prevalence of CVD risk factors continues to rise in Ethiopia despite the inclusion of NCD prevention activities in health extension programmes. Urban and rural communities exhibit low levels of CVD risk perception, and interventions targeting CVD risk factors are inadequate, particularly in rural areas with limited healthcare access [13]. Astudy by researchers at the Armauer Hansen Research Institute (AHRI) confirmed the perception of CVD as untreatable due to limited access to care, a lack of skilled healthcare workers, and religious beliefs [14]. AHRI actively engaged stakeholders, including policy-makers, community members, and healthcare providers, through community advocacy meetings and consultations. Extensive deliberations led to the formulation of recommendations, documented in two issue briefs shared with the Director of NCDs in the Ministry of Health (MOH) in 2022. The incorporation of citizen science in the process strengthened the evidence base for EIDM and paved the way for targeted interventions to address CVD challenges in the local context. One example is the design of a visual aid in the local language for health education by health extension workers. Training sessions were also held to help workers improve their ability to detect and refer high-risk CVD patients, which aided in the identification of previously undiscovered new cases as well as CVD patient flows to nearby health clinics.

The MOH in Malawi hosts a dedicated Knowledge Translation Platform (KTP), which engages policy-makers, researchers, and implementers to facilitate a coordinated approach for evidence generation and use. The ongoing relationship between researchers at the Kamuzu University of Health Sciences (KUHES) and the KTP served as a platform for engagement during the COVID-19 pandemic when there was a growing need for EIDM. The demand for EIDM capacity enhancement across research institutions in Malawi resulted in a COVID-19 policy workshop led by the KTP and co-facilitated by CEBHA+ consortium researchers from KUHES. The KTP and KUHES CEBHA+ teams in collaboration with a multitude of national and international stakeholders, also planned a ‘Building Back Better’ conference in late 2021, which attracted public health professionals, policy-makers, and researchers from different sectors.

CEBHA+ researchers at the University of Rwanda engaged with the MOH, Ministries of Education, Local Governance, Public Services, Rwanda Biomedical Centre, traffic police, and public and private health facilities to mitigate NCD risk factors. COVID-19 mitigation measures, however, negatively affected government interventions such as ‘Friday Physical Activities’ for civil servants and a car-free day for community members. Reduced physical activity and increased risk of NCDs were compounded by low community knowledge of NCDs, leading to concerns about a pandemic within a pandemic [15]. As a result of IKT, the MOH and other stakeholders discussed interventions to be implemented during the pandemic, including animated television sessions, mobile phone applications, and physical attendance at neighbourhood gatherings. Local authorities and community health workers monitored and reported on the implementation. CEBHA+ researchers subsequently prepared four issue briefs on physical activity and joined ministries and private and public institutions to mobilise communities to participate in physical activity and NCD risk screening after pandemic restrictions were reduced.

## Discussion

Developing adequate IKT strategies is key to supporting EIDM, as the case studies in this viewpoint illustrate. By actively involving researchers, policy-makers, practitioners, and community members, IKT fosters collaboration, improves understanding, and facilitates the translation of research findings into evidence-based interventions. This was facilitated in the various contexts by packaging and tailoring research results and their dissemination through trusted platforms such as media, places of worship, and community meetings. CEBHA+ teams also demonstrated that public health interventions should be supported by institutional policies, regulations, and various forms of engagement. Embracing stakeholders who were partners, collaborators, intermediaries, or champions was found to advance advocacy efforts in unique ways, as one partner noted: ‘They sort of have a way of adding a face to the stories and numbers that we (academia) produce.’

The stories in this viewpoint demonstrate that regardless of context, grounding IKT in a deliberate and structured process of stakeholder mapping and engagement is important for value and co-ownership to be nurtured. This was magnified during the COVID-19 pandemic, where the demand from governments in Malawi, Uganda and Rwanda was key to sustainability. Ad hoc engagement through personal networks and opportunities was equally indispensable in the different contexts, but especially in South Africa and Ethiopia, as reported in a previous paper [16]. What is particularly important in these stories is the engagement of stakeholders beyond the health sector where multi-sectoral engagement was key to addressing NCDs.

The participatory approach resulted in robust research outcomes that were contextually relevant, culturally sensitive, and actionable. Implementing an IKT approach, however, also presented challenges. Balancing power dynamics and ensuring equitable participation among stakeholders required ongoing dialogue and capacity building, while sustaining long-term collaboration and securing adequate resources for effective IKT required continuous attention. The embedded knowledge translation platforms in Malawi and at AHRI are excellent examples of planned sustainability that will continue to facilitate the utilisation of research and evidence for use in policy-making.

IKT was also found to be a process and a learning curve that operates at different pace and times for different stakeholders, including the need for tailored training formats adapted to the needs of both knowledge users and producers. Training workshops for policy-makers, for example, need to be flexible and reduce opportunity costs, while workshops for researchers need to cater to and serve immediate project goals.

The ultimate impact of IKT will be assessed in a mixed-methods evaluation across CEBHA+ partners [18]. While this aims to assess how the IKT approach contributed to increased uptake of contextualised research in NCD policy and practice, the process evaluation conducted in tandem aims to shed light on the dose, fidelity, and quality of IKT strategies at each site [17]. Given the complex and politicised nature of policy-making, it will be difficult to assess impact, but the stories certainly illustrate how the call for EIDM and the need to advance progress on the SDGs can be addressed in ways that are both locally relevant and effective.
